# In praise of postgraduate career clinics: Translating health professionals' willingness to engagement

**DOI:** 10.1002/nop2.2113

**Published:** 2024-02-17

**Authors:** T. Redwood, A. Ward, T. Ali, C. Poole, C. O'Dell, D. Rebaudo

**Affiliations:** ^1^ University of Northampton Northampton UK

**Keywords:** advertising strategies, postgraduate education, university infrastructure

## Abstract

**Aim:**

To capture and retain healthcare staff in postgraduate courses relevant to individual career aspirations, service requirements and continuous practice development (CPD) within an English UK university.

**Design:**

Two virtual career clinics for postgraduate practitioners to engage in CPD offers within the university. An online post‐enrolment online survey to explore their experiences of engagement with the university.

**Methods:**

Mixed: qualitative and quantitative methods. Engaging 10 participants attended the career clinics, and 42 participants with an online survey.

**Results:**

The career clinics were well received by participants who mapped CPD requirements and individual career aspirations. The surveys exposed challenges with marketing and enrolment; however, these were mitigated with support. Four recommendations are presented within this paper applicable to the international postgraduate education of all health practitioners.

## 1 INTRODUCTION

In healthcare, continuous professional development (CPD) is fundamental for ensuring frontline staff practice safely and effectively (Manley et al., 2018) and is mandatory amongst UK and international healthcare practitioners such as nurses. Karas et al. (2020) reviewed CPD requirements as mandatory for approximately 1.5 million individuals who are working and registered under 32 healthcare‐regulated titles within the UK. Healthcare professionals are willing and committed to postgraduate education however, they encounter difficulties with engagement (Mlambo et al., 2021). Mlambo et al. (2021) cite some of the barriers to healthcare practitioners' engagement included poor staff levels, heavy workloads, lack of funding, lack of study time and anti‐intellectualism.

Difficulties also include the knowledge of course options, the challenges of full‐time employment often combined with rotational antisocial shift patterns, unclear career advice, confusing postgraduate course pre‐requests and most recently, the Covid‐19 pandemic. The COVID‐19 pandemic has led to the extensive disturbance of medical and health professions education and training (Ahmed et al., 2020). The challenges are exacerbated for those who are ‘trained’ within hospital systems or on the job, for example, paramedics, and are either unfamiliar with universities, and virtual platforms or have not engaged in higher education for long periods of time (Ahmed et al., 2020).

A desk‐based review conducted by Redwood et al. (2021) identified that, on average, universities have 12 healthcare postgraduate courses and 41 CPD courses on offer, highlighting the diversities of postgraduate provision at any one university. Clear pathways are required to enable individuals to navigate their options, which are not always obvious to healthcare practitioners. Mlambo et al. (2021) argued that postgraduate studies could be made more attainable, realistic and relevant. In the current open market, individuals may fulfil their commitments through a variety of mechanisms. This frequently results in healthcare practitioners investing in universities, online platforms or ad‐hoc in‐house healthcare training, which might not meet their requirements, assist in career progression or address service requirements (Redwood et al., 2021). Additionally, universities have developed postgraduate courses and CPD activities with minimal input from their end users, in this case, Healthcare providers, such as the National Health Service, creating more difficulties for healthcare practitioners.

The following paper explores the value of, and lessons learned from undertaking career clinics for healthcare professionals and an online evaluation of current provisions at an English UK University to inform future marketing strategy, as part of an innovation research study, ‘Converting Willingness to Engagement’. The research study was designed to incorporate multiple methods of evaluation for stronger evidence and increased confidence in the results and findings. NHS Healthcare Education England (2023) defines career clinics as opportunities for healthcare practitioners to access a coaching conversation to explore opportunities for development, career moves and transitions.

This article presents the campaign, channel breakdown and top‐performing creatives of the marketing. It concludes with the presentation of recommendations targeting both marketing strategies and the university infrastructure. Ultimately, this research study explored how healthcare practitioner willingness can translate into engagement, using career clinics and how this could be facilitated by postgraduate education providers, such as universities.

## 2 METHODS

The aim of this research study was: To explore postgraduate healthcare provision relevant to individual career aspirations, service requirements and continuous practice development (CPD) within an English UK university.

This research study involved three phases, including an initial focus group with healthcare stakeholders, with these findings reported in a different paper (accepted waiting publication). The delivery of career clinics, a recommendation of this initial phase, for healthcare professionals and a post‐enrolment evaluation with current students on the enrolment process are presented in this following paper. The findings from the career clinics, and the online evaluation, highlight the requirements of effective communication, negotiable websites with accurate information and a key point of contact.

### 2.1 Willingness to engage

Two ‘career clinics’ were publicised via Facebook and Instagram, which were novel ways of marketing to this group by the CPD team, in April 2021. The Facebook and Instagram adverts were aimed at post‐qualifying or postgraduate healthcare professionals, targeted by geographical location within the UK, with an audience scope of health NHS professionals (already working within a hospital or healthcare environment) over the age of 25 years, and with an area of interest in (higher) education. The additional promotion involved emails to known contacts through NHS trust email accounts. The virtual events were open to healthcare practitioners and were developed to address the specific needs of these professionals. Originally the research study was planned to deliver the career clinics face‐to‐face within the six local healthcare Trusts. However, due to the Covid‐19 pandemic and lockdown measures, these were adjusted to virtual drop‐in sessions accessed via email invites to the Microsoft Teams' platform.

The sessions were facilitated by postgraduate university lecturers at different times of the day during the same week to provide a flexible approach for shift‐working healthcare staff. A total of 10, participants engaged in the career clinics. The facilitators wrote reflective notes of each session, kept a record of the number attending and questioned the participants about their sources for the career clinic. The clinics were recorded, and additional facilitator feedback was added about each session. No names or organisational details were stored as part of this reflection. The analysis of the career clinics was undertaken by the entire research team using Braun and Clarke's (2006) six‐stage process of thematic analysis.

### 2.2 Engagement

An online evaluation survey was developed to capture the views of current postgraduate students on their pre‐enrolment and registration experiences, using a combination of open and closed questions. A survey link was shared with newly engaged postgraduate healthcare practitioners and CPD healthcare cohorts from January to May 2021. The aim of the evaluation was to identify the applicants' journey to enrolment. This evaluation was carried out via an Online Survey, with 42 completed responses received. Questions explored how students on the professional postgraduate courses chose their place of study; their reasons for choosing the course, and an evaluation of the registration process. A descriptive data analysis was undertaken using the Statistical Package for the Social Science (SPSS, IBM v26). The findings are presented as percentages of those responding to each question.

## 3 RESULTS AND FINDINGS

### 3.1 Willingness to engage

**FIGURE 1:**
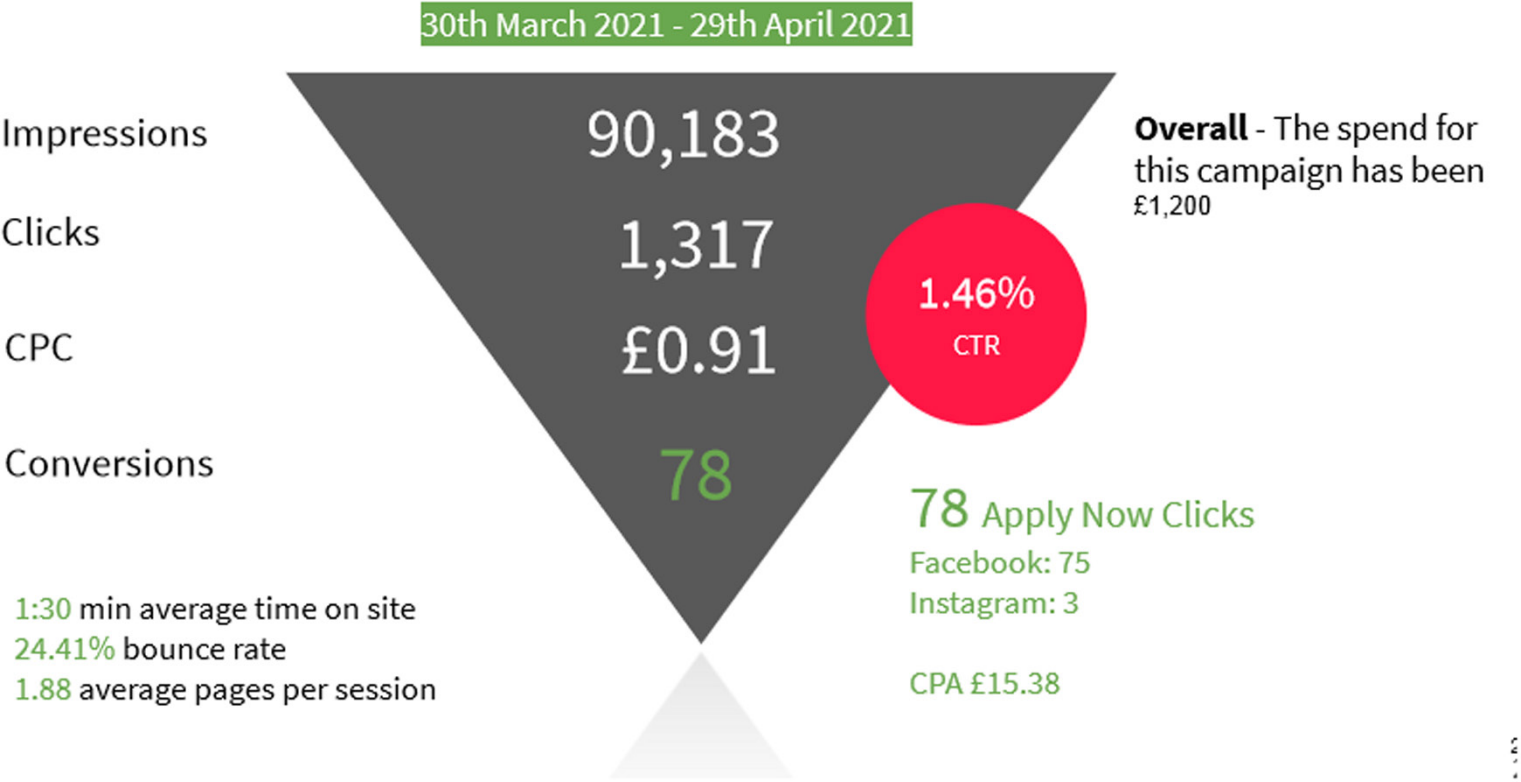
Health professionals career clinics.

**FIGURE 2:**
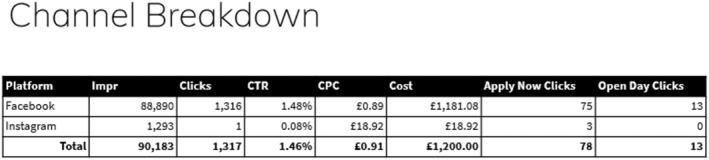
Channel breakdown.

**FIGURE 3:**
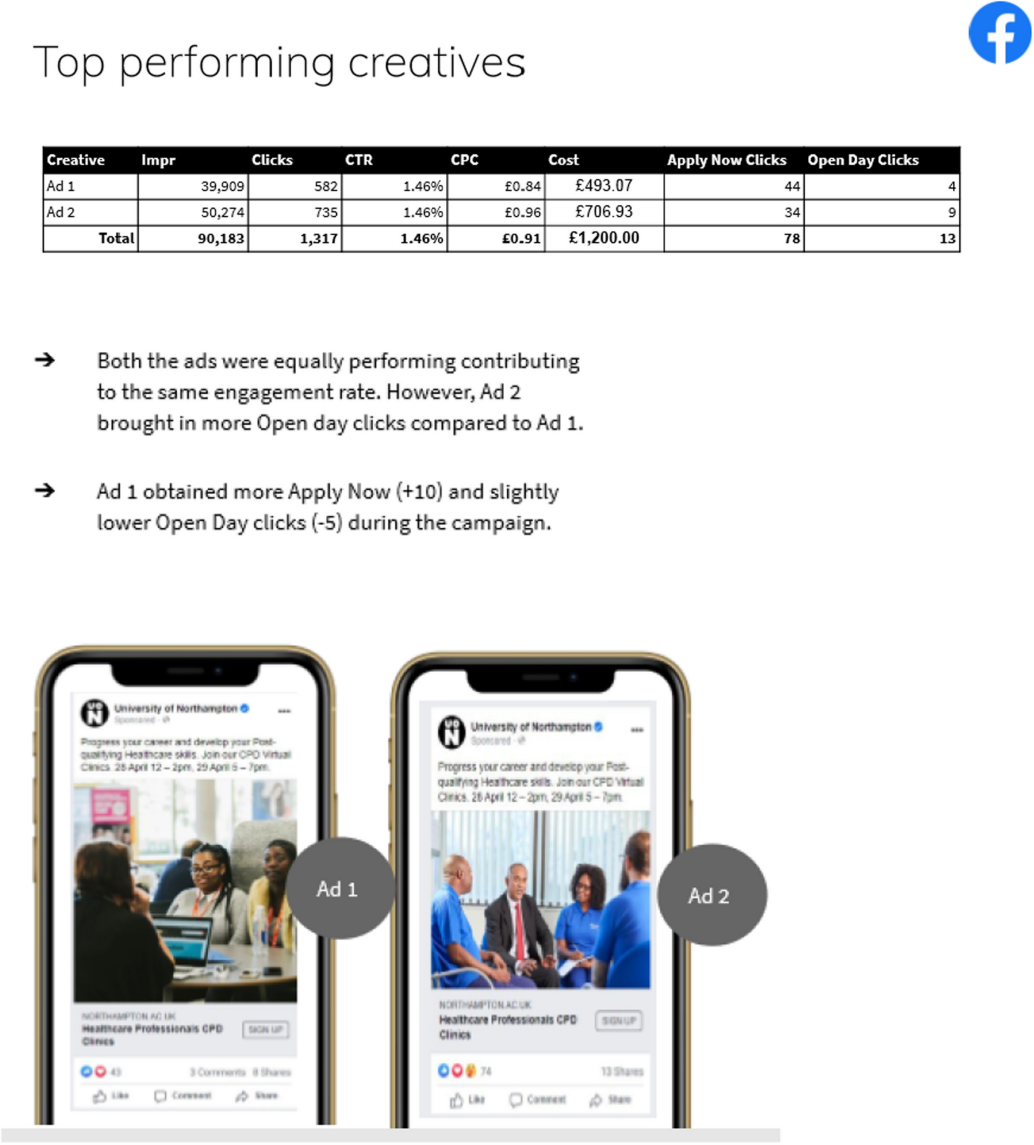
Top performing creatives.

The Facebook and Instagram campaigns of the two career clinics in April 2021 were facilitated by the university and promoted via an agency. The online promotion budget delivered a total of 90,183 impressions, and 1317 clicks, resulting in a CTR of 1.46% (see Figures 1, 2, 3). The Facebook advert resulted in 75 Apply Now clicks and 13 Open Day clicks on the university website. Comparing Facebook against Instagram, Facebook performed better in terms of overall impressions and conversions. Major engagement in the adverts were between the ages of 35–44 followed by 25–34‐year‐old, with the female gender contributing to more clicks.

Of those attending the career clinic, only one reported to have heard about these through Facebook/Instagram advertising. The other nine had learned about the events directly from their healthcare organisation. Although the online advert achieved strong viewings, it only translated into one person attending the Career Clinics. The direct invites via established contacts, therefore resulted in more people attending and a much better conversion rate. The following three figures present these results.

All participants engaged in the career clinics reported their experiences to be both productive and helpful for their individual career aspirations. Participants were able to develop a pathway to attain postgraduate education and meet their CPD requirements.

### 3.2 Engagement

The current postgraduate students who responded to the online survey were demographically divided, with over half (57%) aged 21–29 years, and the majority female (86% female, 14% male). The participants ranged in terms of their professional backgrounds, with 29% being Registered Nurses and 31% from other health professions, e.g., Dentistry, Microbiology, Carers, laboratory technicians and some non‐health professions e.g., humanitarian, zoology and business (see Table 1 for full profile). Finally, 64% came from an organisation other than one of the local healthcare organisations, e.g., Ambulance service, private healthcare providers, London Trusts, or were not in employment.

**TABLE 1:** Participant demographic profile.

	%	No.
Gender		
Female	85.7	36
Male	14.3	6
Other	0.0	0
Total	100	42
Age		
21–29	57.1	24
30–39	21.4	9
40–49	16.7	7
50–59	4.8	2
60 and older	0.0	0
Total	100	42
Profession		
Dietician	0.0	0
Registered nurse (adult, child, mental health, learning disability, SCHPN)	28.6	12
Midwife	4.8	2
Nurse associate	0.0	0
Occupational Therapist	14.3	6
Operating Department Practitioner	0.0	0
Paramedic	9.5	4
Podiatrist	0.0	0
Social work	9.5	4
Physiotherapist	0.0	0
Pharmacist	2.4	1
Other	31	13
Total	100	42

When probed about where they had first heard about their course, 31% (*N* = 13) of those responding reported that the website had been a key source of information, and 21% (*N* = 9) had learned about the course as they were already studying at the university or had been former students. Additional information was provided through friends and family (17%, *N* = 7) and other sources (12%, *N* = 5), such as healthcare job website, personal research and from their employer (5%, *N* = 2) or colleague (5%, *N* = 2).

**FIGURE 4:**
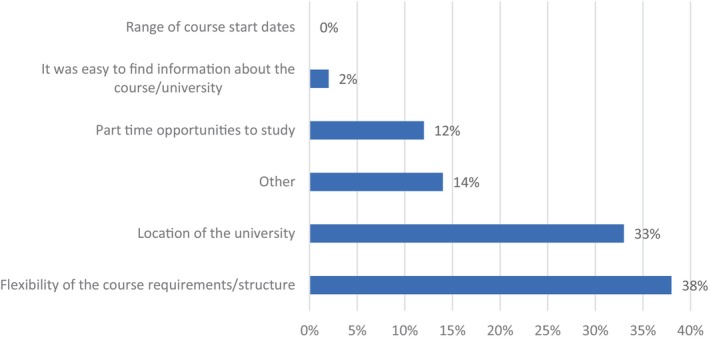
Key factors in deciding to study at the University.

Deciding factors for studying at the University, were the flexibility of the course (38%, *N* = 16), the location (33%, *N* = 14), and the part‐time opportunities (12%, *N* = 5), see Figure 4. Additional considerations included participants who were already studying at the university, and that the fees were affordable (or there was an alumni discount).

When questioned about the key factors for choosing a course, participants identified these as: career development (43%, *N* = 18); continued personal development (24%, *N* = 10); personal interest (19%, *N* = 8); and professional registration requirements (12%, *N* = 5). Participants were asked to comment on whether they were undertaking their course for their professional registration requirements. Those who responded, reported that they had decided on their specific course primarily for career development, however, the following factors were also cited: Proximity to home; Enhance experience; Gain knowledge; To gain professional qualifications; Personal interest; Recommendation from practice educators/managers.

I am already first registrant adult nurse on *** register but have to complete this course in order to be registered as a SCPHN and practice as a health visitor. The university provides the course to individuals recruited and sponsored by local trusts. I had to use this approach as it is the only way to undertake the course and gain qualification needed. (online response 10)

This course is having many connections with the previous course I have done. So, it will be really helpful for professional registration and development. (online response 29)

The participant's stated their choices were due to personal development, wanting to grow as a professional, and to enhance their knowledge and skills. Other factors included: To learn about research methods and gain knowledge of evidence‐based practice; Expand on career opportunities to go into new areas; To gain practical and theoretical knowledge; To gain learning and teaching strategies; and work towards Master's level qualifications.

I am able to use this course for continuous practice development as it will allow me to develop professionally with new skills required for the role of a health visitor. (online response 10)

Participants were asked about their longer‐term career aspirations. The majority cited the key reason as furthering their education to achieve Master's level qualification and/or lead to future PhD study. Participants also engaged to provide qualifications to enter specific careers, for example, in public health and health visiting. Ambitions included expansion into career opportunities abroad. Decisively, the participants reported their aspirations to include developing knowledge; developing research opportunities; providing quality care for people, and providing an evidence base to improve practice and patient care.

Well, being a health worker, this course is a perfect fit for my career growth in fact it helps me to do a medical research, without this course my master's degree is not sufficient to do a medical research. (online response 12)

**FIGURE 5:**
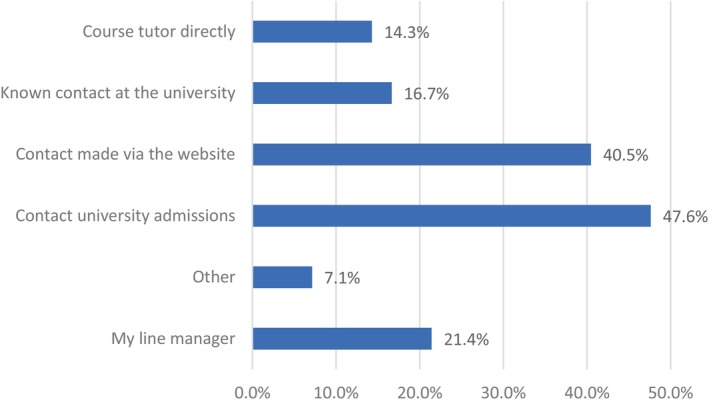
Sources of information about the course.

Forty–eight per cent (48%, *N* = 20) of participants gained information about their course through university admissions, and 41% (*N* = 17) used the website. Participant's contacted line managers, course tutors and a known contact at the university, three other options were also reported, and these were speaking to an agency, a practice development team and attending university open day (see Figure 5). University open days are an opportunity to gather more information about an institution, experience the campus and meet both students and staff.

Participants were specifically asked if they had attended an information event, either at their healthcare trust or via another organisation. Only two respondents (5%) had attended an information event, and one participant reported that this had been ‘really helpful’.

The experience of applying online and the application process was explored with participants. The majority of those responding commented that this had not been too difficult, although they needed patience. They stated it had been ‘flexible’ and ‘straightforward’, although the process was made easier if all the required documents were at hand and they specifically mentioned their previous qualification documentation.

It felt very easy to complete the questionnaire because all instructions and guidelines were given there. (online response 19)

It was not easy or difficult, it can be completed with patience, and it will be good if we have all the necessary requirements documents. (online response 16)

Some participants, however, found the process challenging, stating that it had taken a long time and that the pre‐enrolment and registration website forms were very similar and could be confusing. Some participants reported navigation of the website as being difficult.

It was a little frustrating because if you wanted to go to a page, you had to go through all the pages one by one. There was no menu bar. (online response 26)

Participants were asked about the registration process, 45% (*N* = 19) felt it was easy, and 17% (*N* = 6) thought it was very easy, while 31% (*N* = 13) reported to be neutral and 7% (*N* = 3) indicated it was difficult. When asked to comment about their experiences with the registration process, the participants were concise with their answers. As with the application process, generally, it was reported to be an ‘easy’ and ‘swift’ and ‘seamless’ process. Some challenges were due to late release of grades or Covid‐19 restrictions. Three participants felt the process was repetitive, with details inputted in several places. One participant had challenges locating the correct information online, and two participants mentioned receiving support to complete the process.

Tedious at times. Certain information was asked to be uploaded a few times, despite already being uploaded/asked for. However, this was to confirm sponsorship, therefore may not be applicable to all students. (online response 22)

The Course Tutor was very helpful to iron out some problem in the admission. (online response 40)

Participants were questioned on their experience of navigating the university website. Most stated that it was ‘easy’, ‘very accessible’ and ‘user friendly’. They noted that it provided up‐to‐date information.

The university website is very informative. A new student doesn't need to strive much to know about the university. I really satisfied with the university website. (online response 29)

University platforms were challenging, however, with guidance and familiarity, they were manageable. The process to access different learning tools and register with the library was identified as complex at times.

Initially difficult but I think I understand it now. I find it confusing having so many different learning tools, virtual classroom, pebblepad, padlet etc. (online response 38)

A high percentage of participants (67%) reported they had received guidance from university staff on accessing their course, which may account for why some participants initially reported the experience of registration as ‘easy’. When asked to rate their overall experience in relation to enrolment, 48% (*N* = 20) of participants reported it had been good and a further 24% (*N* = 10) that it had been very good. Just under a quarter (21%, *N* = 9) rated it as acceptable and 7% (*n* = 3) rated it as poor.

The final reflections provided by the participants were positive, and they felt supported. However, aspects could be improved, and these related to providing a more rapid response to confirm submission of the application; More user‐friendly online application; Simplified online registration; and the provision of a phone number or direct contact person to discuss learning and development.

The only suggestion I would make is regarding receiving confirmation on submission of applications to studying a course. I did not receive confirmation until a month later, which made me wonder if it was even received or not. I had to call and find out. I think sending out a confirmation email can easily solve this issue. (online response 15)

## 4 DISCUSSION

Given the nature of health professionals lives, communication and advertising approaches to engage them are challenging. Towers and Towers (2020) explored how postgraduate students made course decisions in a digital era. They discovered decisions to engage involved both rational and emotional choices, highlighting universities need to engage in postgraduates' decision‐making processes prior to enrolment (Towers & Towers, 2020). The findings presented in this paper, under willingness to engage, include the website, career clinics and administration to enrolment procedures. While results, on the engagement experiences, were from the in‐house postgraduate's evaluation survey.

The success of the career clinics, as a pilot, is indicative of an innovative approach which targeted healthcare practitioners and their direct requirements from both rational and emotional perspectives. The career clinics provided a strong and distinctive impression of knowledgeable, accessible university employees willing to dedicate personalised service to aid career development. This is in direct contrast to the historically generic, predominately undergraduate open days that UK universities generate to highlight their prospectuses, campus sites and unique selling points (USPs).

Social media is a fast growing and changing platform for marketing (Galan et al., 2015; Kietzmann et al., 2011). How universities use social media as part of their marketing strategies requires development, especially when exploring how to capture postgraduate students (Galan et al., 2015). In 2021, the most used social network sites were Facebook, YouTube, WhatsApp and Instagram (Statista, 2021), however, Ahmad and Pawar (2021) researched postgraduate students suggesting that professional sites such as LinkedIn may be more suitable platforms for working professionals. Differences in usage are identifiable through age, with young ages tending to prefer platforms such as Facebook and those in older age brackets using LinkedIn (Ahmad & Pawar, 2021). Towers and Towers (2020) state that higher education institutions need to understand engagement and the impact of digital communication use by millennial postgraduate students. In this present research study, the choice of Facebook and Instagram aimed to engage a younger audience, this was supported by the demographic profile of participants from the online survey who were predominantly aged under 40 years. However, X was found to be the most used social media platform for postgraduate purposes (Le Busque & Mingoia, 2021) suggesting this might have been more effective. Trialling marketing in this present study was therefore perceived to be a valuable approach in promoting career clinics. However, the online campaign only converted to one participant. This authenticated the effectiveness of a more traditional approach, the use of email contacts, to promote the career clinics for the engagement of postgraduate health care professionals. These personal contacts with managers provided a more impactful means of marketing and promotion. When reviewing the online surveys with current students, word‐of‐mouth recommendations from friends, family and managers were the key routes to identification of courses. Further review is required to understand postgraduate professionals' engagement with marketing campaigns and the most suitable social media platforms. Identification of a sustainable social media profile for courses and having a ‘buzz’ about these could impact on engagement (Qi & Mitra, 2016). However, other marketing approaches may provide greater benefits as Galan et al. (2015) discovered, social media was not a key source of information in ‘decision‐making’ and therefore made recommendations for stronger links through alumni groups. This resonates with the current findings where key decision factors for current students studying at postgraduate level utilised an alumni discount with the institution. Annual targeted measures with qualified health professionals to re‐engage with their previous university when postgraduate registration requirements are required or future education for career development, is supported by this project's findings.

The 10 participants who attended the postgraduate career clinics represented a considerably larger engagement than those normally attending Open Days across an entire academic year. Staff supporting these clinics reported that they had provided information to potential students about: Financial information and contacts; Advanced practice links with the university; General information about studying at the university. The attending healthcare professionals reported a positive experience in their responses and chat comments. The facilitators perceived those participating as willing to engage in postgraduate courses activities provided by the university. Participants believed future monthly Drop‐in Career Clinics would assist with their engagement and colleagues, into higher education. These findings are indicative of a successful, innovative approach to postgraduate healthcare professionals' requirements. The research team aim to develop these findings not just within the university based in England UK, but to cascade to likeminded universities. The results of the annual Postgraduate Taught Experience Survey (PTES, UK) may provide further insight into the motivations of healthcare professionals for postgraduate study.

The issue of digital literacy was raised through the evaluation questionnaire, as some students found registration and enrolment challenging. Brown et al. (2020) suggested there needs to be a prioritisation for nurses (and perhaps all healthcare practitioners) in the development of digital systems to ensure they are fit for purpose while investing in professional development to attain digital capability. These findings were reflected by NHS Health Education England (2016), which defined digital capability as ‘a necessary skill for living, working, participating and thriving in a digital society’. Similarly, Mlambo et al. (2021) reported participants had enhanced professional confidence resulting from engagement, and conversely, those with poor IT skills believe this a potential weakness. The review by Mlambo et al. (2021) addressed IT concerns as becoming more and more prominent given that more academic programmes are being offered through digital virtual platforms, especially during the post Covid‐19 pandemic. Ensuring potential postgraduate students are skilled to use enrolment and registration systems is vital to converting (willingness) into student places (engagement). Although most current students reported this process to be easy, some experienced challenges of duplication and navigation within the website and more than half received support.

Engaging with complex digital educational platforms at the beginning of an education programme could be facilitated, for example, by YouTube‐hosted videos on the navigation of university systems. Such approaches would further develop the university's presence on social media, building engagement through different routes.

The importance of promoting university websites to enhance engagement with healthcare postgraduate practitioners cannot be underestimated. As nearly a third of the current students in this research study accessed information about courses this way, websites provide a key first‐line resource for potential students. Emphasis on the flexible nature of study is a priority in engaging postgraduate healthcare students who are working full‐time in health settings. Promoting a clear pathway that is easy to navigate and connects with career pathways assists potential applicants with decision‐making. To achieve this, a greater understanding of how to advertise available options to healthcare organisations and cascade this information is required. Emphasis should be on the importance of engaging managers and clear information pathways. Given that 64% of students responding to the survey were from healthcare organisations other than the six local ones, there exists a plausible further reaching marketing plan to target those outside of these core organisations. Plans should be dual purpose, attracting a wide range of health and allied health care professionals in conjunction with capturing existing students to expand conversion from undergraduate to postgraduate. Engagement with the Alumni to facilitate contact with previous students, at selective timeframes, for example, annually, may assist in additional conversions to engagement.

These findings suggest that a personalised approach is required to engage with the University at the postgraduate stage as the digital platform does not fulfil the needs or the personal approach which applicants preferred. A personalised and responsive approach would be an advantage to the university, especially in this competitive market.

The findings from the career clinics, student survey and competitor evaluation presented in this paper potentially have far‐reaching implications and have been developed into the following four recommendations (Table 2) for future impacts on learning and teaching practice.

**TABLE 2:** Recommendations.

Recommendation 1: Provide regular career clinics at an appropriately identified time and day to suit both shift and office‐based healthcare professionals.
Recommendation 2: Identify and highlight a key point of contact within universities for health organisations to streamline engagement.
Recommendation 3: Review postgraduate websites to aid navigation. Provide clear information on costs, potential for modules to build into a programme of study, length of courses and practice implications with increased emphasis on career development.
Recommendation 4: Review the registration and enrolment process for applicants to reduce the duplication of information required and smart navigation capabilities of the forms.

## 5 CONCLUSIONS

This research study aims to explore postgraduate healthcare provisions relevant to individual career aspirations, service requirements and continuous practice development (CPD) within an English UK university.

This paper has provided broad considerations for the future marketing of postgraduate courses within the UK. It has identified challenges of using social media for engagement with health professionals, exploring platforms most suitable and those messages, which are most effective. It became evident that the range of organisations the university engages with is wider than the local previously identified geographic region. Regular review of these organisations would enhance marketing campaigns and widening engagement, locally, nationally and internationally. The website was identified as a key source of information for potential students, and it is important for this to be current and reviewed regularly to ensure information is accurate, informative and attractive. Furthermore, marketing through the alumni may also provide a stronger approach for postgraduate courses, with students already familiar with the campus, staff and systems. Finally, the career clinics themselves should indeed be praised as they proved to be an effective way of meeting with potential healthcare postgraduate students. This provision facilitated, one to one discussion, and tailored information allowing time to speak with dedicated postgraduate staff. The career clinics increased engagement and connection with health professionals by facilitating personal connection.

## AUTHOR CONTRIBUTIONS

All authors contributed to this article under the ICMJE recommendations.

## CONFLICT OF INTEREST STATEMENT

The authors declare no conflicts of interest.

## ETHICS STATEMENT

The research study was undertaken between September 2020 and June 2021 following a successful review by the relevant faculty's Research Ethics Committee (REF: FHRECHEA000252). Participants voluntarily engaged and were fully informed of the aims of the project. Consent was gained at the outset of all data collection methods.

## Data Availability

Data Policy link to PURE account linked to original report and first article in submission with Nursing Open.
